# Exploring the design and utility of an integrated web-based chatbot for young adults to support healthy eating: a qualitative study

**DOI:** 10.1186/s12966-023-01511-4

**Published:** 2023-10-04

**Authors:** Lee M Ashton, Marc TP Adam, Megan Whatnall, Megan E Rollo, Tracy L Burrows, Vibeke Hansen, Clare E Collins

**Affiliations:** 1 School of Health Sciences, College of Health, Medicine and Wellbeing, University of Newcastle, 2308 Callaghan, NSW Australia; 2 School of Education, College of Human and Social Futures, University of Newcastle, 2308 Callaghan, NSW Australia; 3 Active Living Research Program, Hunter Medical Research Institute, 2305 New Lambton Heights, NSW Australia; 4 Food and Nutrition Research Program, Hunter Medical Research Institute, 2305 New Lambton Heights, NSW Australia; 5 School of Information and Physical Sciences, College of Engineering, Science and Environment, University of Newcastle, 2308 Callaghan, NSW Australia; 6 Curtin School of Population Health, Faculty of Health Sciences, Curtin University, 6845 Perth, WA Australia; 7 School of Medicine and Public Health, College of Health, Medicine and Wellbeing, University of Newcastle, 2308 Callaghan, NSW Australia

**Keywords:** Young adults, Qualitative research, Nutrition, Healthy eating, Chatbot, Behaviour change, Diet, Website

## Abstract

**Background:**

There is a lack of understanding of the potential utility of a chatbot integrated into a website to support healthy eating among young adults. Therefore, the aim was to interview key informants regarding potential utility and design of a chatbot to: (1) increase young adults’ return rates and engagement with a purpose-built healthy eating website and, (2) improve young adults’ diet quality.

**Methods:**

Eighteen qualitative, semi-structured interviews were conducted across three stakeholder groups: (i) experts in dietary behaviour change in young adults (n = 6), (ii) young adult users of a healthy eating website (n = 7), and (iii) experts in chatbot design (n = 5). Interview questions were guided by a behaviour change framework and a template analysis was conducted using NVivo.

**Results:**

Interviewees identified three potential roles of a chatbot for supporting healthy eating in young adults; R1: improving healthy eating knowledge and facilitating discovery, R2: reducing time barriers related to healthy eating, R3: providing support and social engagement. To support R1, the following features were suggested: F1: chatbot generated recommendations and F2: triage to website information or externally (e.g., another website) to address current user needs. For R2, suggested features included F3: nudge or behavioural prompts at critical moments and F4: assist users to navigate healthy eating websites. Finally, to support R3 interviewees recommended the following features: F5: enhance interactivity, F6: offer useful anonymous support, F7: facilitate user connection with content in meaningful ways and F8: outreach adjuncts to website (e.g., emails). Additional ‘general’ chatbot features included authenticity, personalisation and effective and strategic development, while the preferred chatbot style and language included tailoring (e.g., age and gender), with a positive and professional tone. Finally, the preferred chatbot message subjects included training (e.g., *would you like to see a video to make this recipe?*), enablement (e.g., *healthy eating doesn’t need to be expensive, we’ve created a budget meal plan, want to see?)* and education or informative approaches (e.g., “*Did you know bananas are high in potassium which can aid in reducing blood pressure?”*).

**Conclusion:**

Findings can guide chatbot designers and nutrition behaviour change researchers on potential chatbot roles, features, style and language and messaging in order to support healthy eating knowledge and behaviours in young adults.

**Supplementary Information:**

The online version contains supplementary material available at 10.1186/s12966-023-01511-4.

## Background

Searching online for health information has accelerated in line with advances in technology and social media uptake [1–3]. This is especially true among youth, including adolescents and young adults [3]. There are numerous advantages to accessing health information online, including information immediacy and anonymity, which are especially appealing to younger generations [3]. A recent review of digital health technology use among young people found that websites and search engines were used more commonly, whereas social media, self-tracking apps and wearable devices were experimented with but not typically used long-term [3]. While the volume and breadth of health information available online was seen as positive, the review also identified this as a barrier in terms of ability to navigate through information [3].

Chatbots offer a more personalised approach when seeking health information online (e.g., booking a consultation or asking health-related questions) [2]. Chatbots are software applications with a user interface to allow communication via text or text-to-speech [2]. Chatbots can be either constrained (i.e., users select pre-programmed responses in the chat), or unconstrained using natural language input, which allows free text or speech [2, 4]. The use of chatbots in health contexts is increasing [2, 4, 5]. A 2018 systematic review of studies using unconstrained chatbots for health-related purposes identified 17 studies, with most targeting patient support related to mental health [2]. Included studies evaluated user experience and chatbot technical performance, as well as patient adherence to self-care practices and diagnostic performance in relation to health conditions, including mental health, asthma, hypertension, type 2 diabetes, breast cancer, obstructive sleep apnoea, sexual health, pain monitoring and language impairment. Overall, studies reported high user satisfaction and usability, moderate to high recognition accuracy and successful task completion of the chatbots, with high specificity of chatbots for diagnosis of depression, and sleep problems [2]. One randomised controlled trial also reported a significant reduction in depression symptoms in young adults following a two-week intervention using self-help content provided via chatbot [6].

Further, studies have demonstrated potential for chatbot technology to promote health behaviour change [4, 5]. For example, a 2021 systematic review of studies using chatbots to improve physical activity, dietary change and weight loss included nine studies in adolescents and adults [4]. Five of seven studies targeting physical activity found positive results, including increases in daily step count, moderate-to-vigorous physical activity levels, and completion of physical activity goals. Only four studies had targeted dietary intake, including two with positive results for intention to reduce processed meat intake and adherence to a Mediterranean diet. Only one study (non-RCT) had targeted weight loss and found a significant reduction at 12-weeks (-1.3 kg, *p* = 0.01). Most studies used constrained chatbots and recruited adults aged 18 years or older, rather than at specific stages of adulthood. In addition, the review found few studies had utilised a theoretical framework to design and evaluate the chatbot technology and highlighted the importance of doing this in future studies to help understand the underlying principles driving participants’ motivation, engagement, and behaviours [4]. Another recent systematic review and meta-analysis found chatbot interventions (n = 4 studies) to be efficacious in improving fruit and vegetable consumption by 1 serving per day (95% CI: 0.30, 1.68) [7]. Although this review was not specific to young adults (median age of 44 years), it highlighted the need for chatbot interventions to be designed with longer-term implementation in mind and collaboration with different stakeholder groups (e.g., research/tech industry/health care system partnerships).

Despite the increasing number of chatbots designed for use in health contexts, limited research has been conducted regarding chatbots for nutrition promotion, or those targeted and tailored to young adults. This is a major gap given that young adults commonly seek health information online [3]. Further, young adults should be a priority group for nutrition interventions given current unhealthy dietary patterns, and hence elevated risk for diet-related chronic disease [8–10]. In a global assessment of diet quality from 187 countries, young adults aged 20–29 years had the lowest diet quality scores (indicating the poorest eating habits), compared with all other adult age-groups [8] This indicates that they have higher intakes of energy-dense, nutrient-poor foods (e.g., processed meats and sugar sweetened beverages) and lower intakes of nutrient-rich foods (e.g., fruit and vegetables, and wholegrains) [8]. Young adults also experience unique life stage transitions related to changes in social relationships, living arrangements and financial status which can impact on their eating habits and other lifestyle behaviours [11, 12]. As such, targeted approaches are likely to be needed to understand how best to support and motivate young adults to eat more healthily. A recent review of chatbots for nutrition and physical activity among adolescents found co-design with the population group to be vital to ensure the technology is feasible and acceptable to the targeted population [13]. Qualitative interviews can play an important role in knowledge generation [14] and provide in-depth exploration on a topic for which there is limited empirical evidence [15]. To the authors’ knowledge, there has not been previous qualitative research exploring the potential design and utility of a chatbot to support healthy eating in young adults.

Therefore, the current research aims to address the gaps in the literature and build on the recommendations from previous reviews within the field, including the need for chatbot co-design with the target population [13], utilisation of a theoretical framework to inform design of the chatbot [4] and development of a chatbot with longer-term implementation in mind [7]. As such, the aim of this research was to conduct qualitative interviews with (1) experts in dietary behaviour change in young adults, (2) young adult users of a healthy eating website, and (3) experts in chatbot design, regarding how chatbots could be designed to:

1. Increase young adults’ return rates and engagement with a purpose-built healthy eating website.
2. Improve young adults’ diet quality over time.

## Methods

### Study design

This was a qualitative study, with a focus on the exploration stages of design science research methodology [16]. The conduct and reporting of this paper adhered to the guidelines outlined in the consolidated criteria for reporting qualitative research (COREQ) [17] (See Additional File 1). Ethical approval was obtained from the University of Newcastle Human Research Ethics Committee (HREC-2018-0512).

### Framework

This study used a hybrid approach, using both deductive and inductive reasoning [18–20]. Deductive reasoning is a top-down approach (from theory to data), whereas an inductive approach is a bottom-up method aimed at finding relationships between themes that emerge from participants’ opinions [21] and generating knowledge to inform design of chatbot systems in this context [22].

### Deduction: building on the behaviour change wheel

**Fig. 1:**
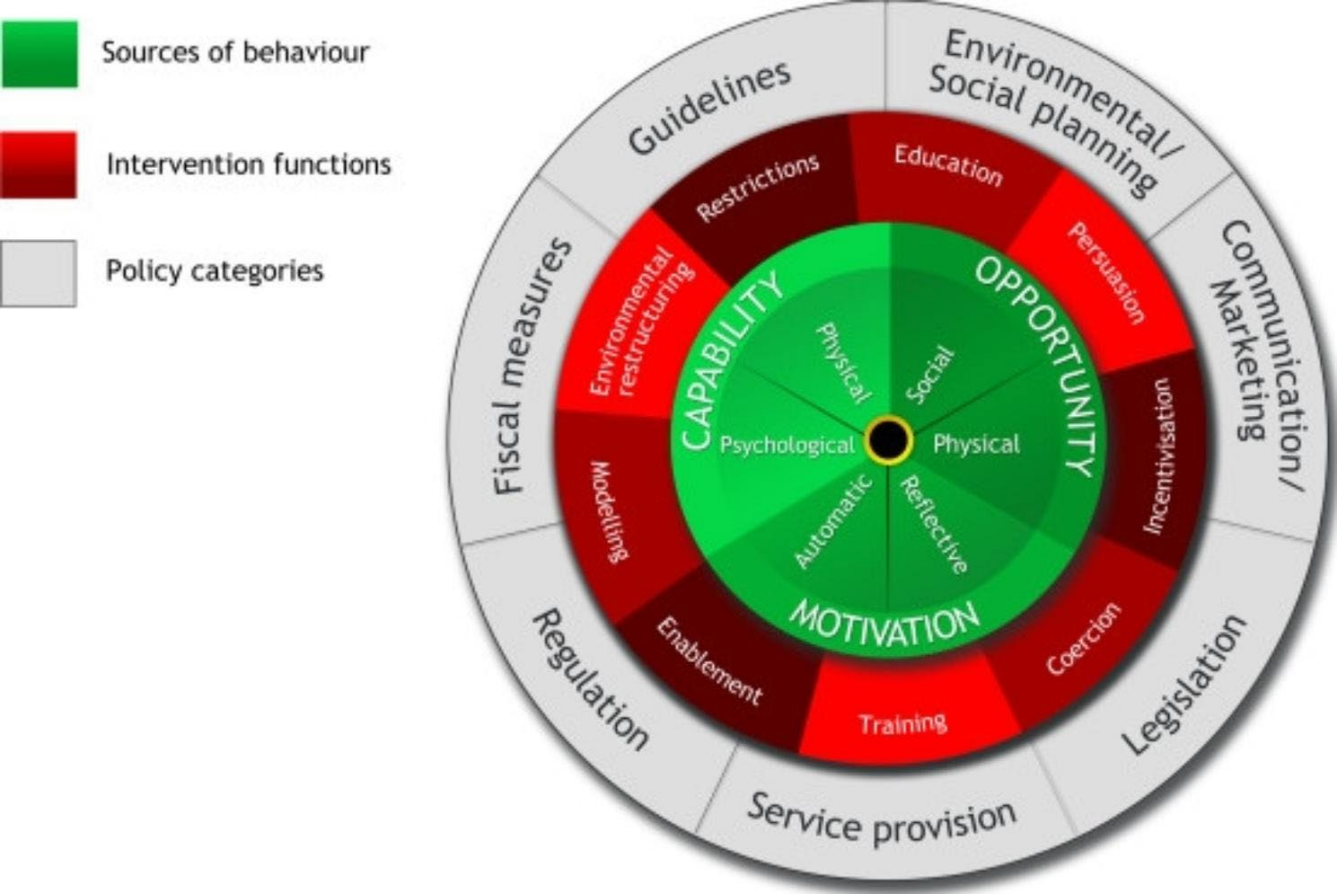
The Behaviour Change Wheel. Image obtained from: Michie S, van Stralen MM, & West R (2011), The behaviour change wheel: A new method for characterising and designing behaviour change interventions. *Implement Sci.***6:42** [23]

For the deductive approach, the behaviour change wheel (BCW) [23] was utilised to frame questions for the interviews (Fig. 1). The BCW recognises that behaviour is part of an interacting system involving the capability, opportunity, and motivation components which can be utilised when trying to achieve behaviour change. Interventions need to change one or more of these capability, opportunity, and motivation components in such a way as to reconfigure behaviours into new habits and minimise the risk of reverting to old behaviours.

### Interviews

The interview questions focused on exploring i) capability, opportunity, and motivation factors believed to be most influential on young adults’ eating behaviours (green circle in the BCW). In the current context, ‘behaviours’ are defined as those aimed at:

i. Increasing young adults’ return rates and engagement with a purpose-built healthy eating website (https://nomoneynotime.com.au/ [24]).
ii. Improving young adults’ diet quality over time.

Questions were developed deductively and focused on selected intervention functions (red circle in the BCW) to facilitate design of effective strategies to improve the behaviours above, while generation of open codes was inductive and derived from interview transcripts. Additional file 2 provides details of the interview guide.

### Participants, recruitment & setting

Purposive sampling was used to identify the following three stakeholder groups for interviews:

1. **Experts in dietary behaviour change in young adults***(referred to as ‘dietary experts’ from herein*).
2. **Current users** of a purpose-built healthy eating website (https://nomoneynotime.com.au/ [24, 25]) and aged 18–35 years (*referred to as ‘current website users’ from herein*).
3. **Experts in chatbot design ***(referred to as ‘chatbot experts’ from herein*).

Participants from stakeholder groups one (dietary experts) and three (chatbot experts) were considered experts based on their experience and/or relevant research outputs and were identified through (i) previously established contacts (ii) a search on Google and Google Scholar and (iii) exploring reference lists of relevant research [2, 4, 26]. Participants from stakeholder group two (current website users) were purposively selected from a database of users who had signed up to an account on our purpose-built healthy eating website (‘No Money No Time’: https://nomoneynotime.com.au/ [24, 25]) and answered “yes” to the following question *“Do you agree to be contacted about future studies from the University of Newcastle”* when completing the embedded brief, validated dietary quality assessment tool (Healthy Eating Quiz) [27–29].

All individuals were contacted via email from a member of the research team with an information statement to seek their interest and consent for participation in the study. A total of 123 invitation emails were sent (seven emails to dietary experts, 80 emails to current website users and 36 emails to chatbot experts). The information statement outlined research personnel involved in the study and reasons for conducting the research. Participation was entirely voluntary, and participants were required to have English proficiency, sufficient to engage in an interview of up to 60 min. Interested individuals completed an online eligibility screen and were at least 18 years of age, while those in stakeholder group 2 (current website users) were required to be between 18 and 35 years, consistent with the ‘No Money No Time’ target audience. Demographic characteristics for all groups were collected via online survey during the eligibility survey and included sex, age, country of residence, employment status and main occupation field (see Supplementary Tables 1, Additional file 3 for details of the participants’ backgrounds). Interviews were conducted online using Zoom software (Zoom Video Communications, Inc. San Jose, California, USA) with the audio recorded and transcribed verbatim. Only the interviewer and participant were present during interviews. The final data set included 18 interviews. All participants received a $25 gift voucher as reimbursement for their time taking part in the interviews.

### Data collection

Interviews were conducted between 25th March 2021 and 24th August 2021 and lasted on average 31 min (range: 18 to 52 min). All interviews were conducted by a female researcher of similar age to the current website users’ group, who was an Accredited Practising Dietitian and PhD student with previous experience of conducting qualitative interviews. No relationship between the interviewer and participants was established prior to the study. All participants provided written informed consent.

Prior to interview commencement, participants were sent an information statement explaining the interview topic and this was reiterated by the interviewer at the start of each interview. The interviewer also ensured participants were familiar with the definition of a chatbot and its capabilities. Specifically, the interviewer defined a chatbot and images were shared to indicate examples of closed and open chatbot conversations unrelated to healthy eating (See Additional file 2).

The interviews were semi-structured with a total of seven questions asked. Probes were used to clarify and further explore topics (see Additional file 2 for a copy of the interview guide). Furthermore, to elicit in-depth responses to the following question: “*What aspects of content and style do you think are important for a chatbot to use when conversing with young adults about healthy eating?”* the interviewer shared a recently developed taxonomy of social cues for chatbots [26] (See Additional File 2). Similarly, for question: *“What type of messaging would be more persuasive for young adults?”* participants were provided with a table outlining different message types and related examples (See Additional File 2) based on BCW intervention functions. During the interviews field notes were not taken. At the end of the interview, participants were asked to provide any additional comments that may have been missed.

### Data analysis

A template analysis was conducted by an independent qualitative researcher using NVivo Version 12 (QSR International Pty Ltd, 2018 [30]) to assist with the organisational aspect of the analysis. Template analysis [31, 32] is a form of Thematic Analysis [33] deemed particularly useful in instances where researchers wish to apply a high *a-*priori structure to the analysis, which in this case was the behaviour change wheel.

During the initial stage of data immersion, three transcripts from each stakeholder group were read in detail to gain familiarity with the data in relation to the research questions. A list of tentative *a-*priori themes was identified based on discussion with one of the authors (LMA), which aligned with the BCW components. Coding of the data then commenced with codes being placed within the template. This phase was highly iterative with the template flexible, to allow for development of a thematic structure that could accommodate data not aligning with the *a* -priori themes. This approach is consistent with the steps suggested for Template Analysis [31, 34], where *a*-priori themes are identified in advance or concurrently with code generation, and that these are necessarily tentative and open to refinement [31].

Based on coding of several transcripts from each participant group, an initial coding template was developed which was then applied to the remainder of transcripts in each group. During this phase of the analysis, the template was further refined to allow for a comprehensive and hierarchical representation of the data in relation to the research questions. A final version of the template was applied to the entire dataset. Themes were identified and named to most accurately depict the meanings captured within. Verbatim quotes from participants which vividly represented the themes were extracted and presented throughout, accompanied by participant group and ID. Data collection and analysis did not occur concurrently. As the aim of the study was to report on the viewpoints of key informants regarding the potential utility of a healthy eating chatbot, with analysis involving a largely deductive process (i.e., codebook analysis), saturation was measured as the extent to which the derived themes were adequately represented in the data [35–37]. Data saturation was confirmed during data analysis. Specifically, there was repetition in the data (data saturation), the generation of new codes slowed down significantly (inductive thematic saturation), and the data obtained appeared to represent the themes adequately (*a* priori thematic saturation).

## Results

Table 1 summarises participant’s demographic information by stakeholder group. In summary, a total of 18 participants, aged 29.9 ± 8.0 years took part in the interviews. The majority were female (72%), residing in Australia (67%), currently in paid employment (61%) and worked in the health and medical field (33%). Supplementary Table 1 in Additional file 3 provides more detail on participants’ backgrounds and experiences.

**Table 1:** Participant demographics by stakeholder group

	Dietary experts (*n* = 6)		Current website users (*n* = 7)		Chatbot experts (*n* = 5)		Total (*n* = 18)	
	n	%	n	%	n	%	n	%
**Female**	6	100%	6	86%	1	20%	13	72%
**Country of residence**								
* Australia*	6	100%	5	71%	1	20%	12	67%
* Germany*	0	0%	0	0%	2	40%	2	11%
* Netherlands*	0	0%	1	14%	0	0%	1	6%
* New Zealand*	0	0%	0	0%	1	20%	1	6%
* Poland*	0	0%	1	14%	0	0%	1	6%
* USA*	0	0%	0	0%	1	20%	1	6%
**Employment status**								
* Student*	2	33%	5	71%	0	0%	7	39%
* Paid employment*	4	67%	2	29%	5	100%	11	61%
**Main occupation field**								
* Health and medical*	5	83%	1	14%	0	0%	6	33%
* Education*	0	0%	1	14%	2	40%	3	17%
* Science*	0	0%	0	0%	3	60%	3	17%
* Engineering*	0	0%	2	29%	0	0%	2	11%
* Hospitality, travel & tourism*	0	0%	1	14%	0	0%	1	6%
* Management*	0	0%	1	14%	0	0%	1	6%
* Other or not applicable*	1	17%	1	14%	0	0%	2	11%
	**Mean**	**SD**	**Mean**	**SD**	**Mean**	**SD**	**Mean**	**SD**
**Age (y)**	29.8	5.5	23.2	1.7	39.4	6.1	29.9	8.0

### Utility of a chatbot to support healthy eating in young adults

**Fig. 2:**
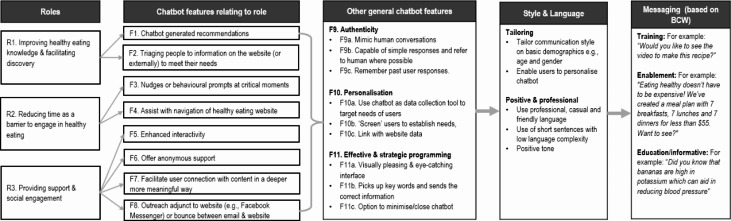
Overview of potential chatbot roles, features, style and language and messaging to support healthy eating

An overview of common themes in relation to the potential roles a chatbot could play to support healthy eating and associated chatbot features, other general chatbot features and preferred style, language and messaging is presented in Fig. 2 and Supplementary Tables 2–5 in Additional file 3, with detailed information provided below. The presentation of results below aligns with the structure for Template Analysis, with main headings relating to the questions asked as the deductive ‘overarching themes’, and the sub-ordinate groupings are then the inductively derived sub-themes.

### Potential roles of a chatbot to support healthy eating

#### R1. Improving healthy eating knowledge & facilitating discovery

All stakeholder groups frequently discussed the potential for chatbots to improve young peoples’ knowledge of healthy eating. Specifically, mention was made of using the chatbot to initiate the conversation based on the user’s browsing behaviour to generate recommendations for content [F1. Chatbot generated recommendations], which may be of interest or able to facilitate “discovery” of new (unintended) content;

*“…maybe you have recipes there and people visit that section of the website and you can open the chatbot and say, “Hey I see you are looking for recipes with… Here’s some of my favorite suggestions. If you want to learn more click here…” (Chatbot expert CE01)*.

*“…you could have the chatbots saying like, let’s say again, you’re looking at like, healthy, vegetarian recipes or something like on the chatbot and it comes up with a recipe. But then it might say to you, “oh, now that you looked at, would you like to see also blah” … It could kind of sneak in, introduce some other good topics that you think might be of interest or that you might want to just promote. (Chatbot expert CE02).*

Furthermore, several dietary experts mused on the concept of chatbots as a means of triaging young people to specific information on the website (or externally) which could address current needs [F2. Triaging people to information on the website (or externally) to meet their needs];

*“…they could pinpoint someone’s lack of knowledge or lack of skills and then maybe direct towards that resource that might help them fill that gap”. (Dietary expert DE05).*

#### R2. Reducing time as a barrier to engage in healthy eating

The barrier relating to lack of time was also viewed by many as being potentially addressed by chatbots, with the use of nudges or behavioural prompts that could be triggered to support people at critical moments (e.g., at a time when they are more likely to order an unhealthy takeaway option) [F3. Nudges or behavioural prompts at critical moments];

*I think if the chatbots there to maybe give some nudges or behavioural prompts or provide some recipes in real time to be a real advantage for young adults that are busy or think that they don’t have time to look at recipes or potentially get home and they’re too busy and they forget and then end up ordering out or getting something delivered. (Dietary expert DE03)*.

Also, it was perceived that chatbots could play a role in assisting users to navigate the information on various pages within a healthy eating website when a conversation is initiated by the user [F4. Assist with navigation of healthy eating website]. This could help address time barriers for users, as well as an avenue for users to access healthy eating knowledge (i.e., Q & A).

*“I think it would be really good if, if I asked a question and it took me exactly to that spot for my answer.” (Current website user U01)*.

#### R3. Providing support and social engagement

The interactivity inherent in using chatbots was considered important for young people [F5. Enhanced interactivity];

*“The ones that are available from government or public health organisations are just pretty static and like text-based boring…they’re all written at a university level. Just having that extra elements on the pages and making them more interactive I think is a really positive step”. (Dietary expert DE04)*

While chatbots were considered to add a friendly and approachable element to interaction within a website, the applicability of chatbots to provide anonymous support [F6. Offer anonymous support] was raised by dietary experts and chatbot experts as an important feature, as this might prevent some feelings of embarrassment and shame associated with particular topics and questions ‘discussed’.

*I guess the first thing that came to mind was that I think both chatbots and conversational agents offer a viewer of secrecy, the same type of viewer secrecy that we have when we search for random things on Google. I think there is quite a bit of stigma when it comes to certain, for example, types of eating disorders. The first thing that came to mind is that something like this would perhaps make it safer for some young adults to get information, get feedback with regards to some sensitive topics related to healthy and unhealthy eating*. (Chatbot expert CE03)

However, there is need for built-in response capability to deal with disclosure of potentially sensitive topics, such as eating disorders. If such capabilities could not be assured, it was advised that the conversation should be designed to be of a closed nature to limit discussion of personal issues. However, irrespective of what approach was chosen, participants emphasised that a clear goal of the chatbot must be established prior to design, and whether it would serve a purely operational purpose or a “social” purpose, capable of conversational chatting.

Furthermore, a chatbot was also seen to facilitate user connection with content on a deeper, potentially more meaningful level [F7. Facilitate user connection with content in a deeper more meaningful way], because of the “social” conversational aspect;

*“There’s something I guess, because we are social creatures, there’s something about having that social interaction through conversation that brings out, that activates a different part of the way that we think; and I guess opens up people to listen in a different way than, you know, reading something on a website or watching a PowerPoint presentation or a video does.” (Chatbot expert CE05)*.

"*I really do think that to trigger behaviour change, you need support, you need a network, and you need a good sense of community. I think technology can definitely provide those as well. And so rather than having a standalone chatbot that a person interacts with, I think that in order to trigger behaviour change, you would probably want the chatbot with some social aspect, whether it’s comparison, whether is getting encouragement and support from your social network, and that’s connected in some way to the chatbot*. (Chatbot expert CE03)

Finally, there were calls for outreach as an adjunct to the website, including an external chatbot to enable greater user reach from platforms they are more likely to use (e.g., chatbot on Facebook Messenger) and the potential for a “bounce” between website chatbot and email [F8. Outreach adjunct to website (e.g., Facebook Messenger) or bounce between email & website];

*They don’t need to go on a website or look for some information. The information comes to them.* (Chatbot expert CE01)

*If you aim for engagement, then you need to use one of the channels that people use anyways.* (Chatbot expert CE01)

*“You have maybe somehow our connection to this machine because you’re having a chat. And when the bot at the end of your chat asks you, “Hey, that was nice, I would like to stay in touch with you. Could you please … if you want to give me your email and I will send you interesting content”. And this is one way that companies use it to, to get email addresses. And maybe this could also be interesting that the bot is not just one way to increase engagement within the bot, but also to recruit people who are interested for, for these automated emails. And then in the emails you can say, “Hey, we’ve recently updated our chatbot. It now has interesting recipes, come here visited again so you can try to…”. (Chatbot expert CE01).*

### Other general chatbot features to support healthy eating

#### F9. Authenticity

Authenticity was considered one of the most salient chatbot features by each stakeholder group. Specifically, dietary experts mentioned that a chatbot, in order to potentially bring about behaviour change through increased motivation, needed to mimic human conversation as much as possible and allow for open conversations;

*“At the motivational level…it depends really on I guess the individual, as to whether a chatbot can increase motivation and whether it comes through is genuine and authentic, cause you’re not really talking to a real person. Again, [it depends on] the sophistications in technology”. (Dietary expert DE03)*

Users also felt it was quite important that the chatbot had human-like qualities and allowed for more personal interactions;

*“…I know sometimes on websites they have like a little human, like a graphical person that pops up, like a little animation and it makes it a little bit more personable” (Current website user U02)*.

One user preferred the option for questions to be answered by a human when the chatbot could not readily locate the answer or understand the question. The need for human back-up is particularly important given the complexity of questions around healthy eating and potential impact on people’s health.

One chatbot expert raised the potential and suggested an advantage of such ‘hybrid’ chatbots which would be capable of simple responses only, but with a referral to a human contact where needed;

*“This is where you have a chatbot as a single contact. And if the chatbot doesn’t know how to move on, it hands over to a human. But this is also something which I could imagine in this foods context where you have some person that really gives you personal advice in case you don’t trust the chatbot, or you want to talk to a human. So that could be a feature where you just have a phone call with someone or you hand over to an instant message chat with real human”. (Chatbot expert CE04)*

#### F10. Personalisation

In terms of personalisation, dietary experts and chatbot experts raised the importance of the chatbot being able to target the needs and characteristics of its users. Given the complexities around healthy eating, it was deemed desirable for it to involve collection of brief data from users to gain an understanding of their current behaviours so that this information could then be used to generate more tailored responses or personalised “interventions” and information;

*“If there’s some way of personalising it a bit to the individual by perhaps asking them a few of those more closed ones on the left-hand side, to get some information about them to then be able to answer the questions in a more personalised way. I think that would make it more engaging and useful for young adults as well”. (Dietary expert DE02)*

*“The more you know about the user, the better you can trigger behaviour change. And that is why this data collection is still super important”. (Chatbot expert CE04)*

Additionally, one user shared ideas as to the aspects of personalisation that, for them, would be ideal;

*“So maybe if there was certain foods that you liked or didn’t like, or if you’re vegetarian, or an amount of time that you wanted to spend cooking. The amount of money you wanted to spend on cooking for the day. Maybe how many meals you cook a day or a week or whatever”. (Current website user U04)*

It was suggested by chatbot experts that screening or information gathering from users was a cornerstone of providing personalised content and advice, as well as optimising the potential for continual engagement. Options were raised of embedding this in the initial interaction in terms of establishing users’ goal of the interaction and their readiness for change;

*“The screening could be something really simple as first as like “what are you here for today?” kind of thing and then it has like a few different options… so as sort of a screening system that I guess identifies that person’s maybe stage of change or where they’re at, and then that would then influence whether the bot has like a negative or a positive tone or a mixture of both”. (Chatbot expert CE02)*

However, discussions by chatbot experts raised the potential for personalising chatbot engagement without the need for data collection from users, but based solely on website engagement;

*“If you see that the person is on the website in the section where you talk about sugar, and then there’s the chatbot pops up and tells you, “Hey here’s some cool information, you didn’t know about sugar”. And I think this is also personalization based on what the user did, but you don’t have to know his or her habits”. (Chatbot expert CE01)*

#### F11. Effective and strategic development

From all participants, it was perceived particularly important that the chatbot would have a visually pleasing and eye-catching interface with the use of avatars or animations, and also have the option to close/minimise the chatbot if required.

*I know sometimes on websites they have like a little human, like a graphical person that pops up, like a little animation and it makes it a little bit more personable.* (Current website user U02)

*So if it’s like show up only once and then you can like minimise that or open it again, or, you know, there is an option to open it or close, but it’s not like it’s going to be showing up every five minutes or something. *(Current website user U06)

Indeed, effective development was seen as very important, as many users shared frustration around previous encounters with chatbots that were poorly designed, such as not picking up on keywords, sending users off to wrong information, or infinite loops.

*“I think what deters me is when it doesn’t give me the answer that I’m after and then it just keeps reverting back to the main menu instead of offering more assistance.”(Current website user U02)*.

This was also reiterated by chatbot experts:

*“This is a very important factor that you do not open up … And sometimes you may allow for open answers, but most of the successful chatbots predefine the structure of the dialogue to some degree. And that is what I definitely also would recommend to you to keep the dialogue simple and also [to have] partially predefined responses.” (Chatbot expert CE04)*.

### Chatbot style and language

#### Tailoring

It was considered very important that the language and style were tailored specifically to a young adult audience and that responses/messages should be developed in close collaboration with young people to ensure correct and appropriate use of generational-specific terms and symbols. Tailoring chatbot communication style to users was also reported by chatbot experts to be important in terms of improving engagement. With personality differences in communication style being a difficult aspect to assess outside of more extensive data collection, one chatbot expert recommended adaptation of a generic communication style on the basis of key demographics such as age and gender;

*“We did some research on personality and chatbots. And we looked at how different personalities of users may be influenced if the chatbot is talking more dominant or less dominant with the user. And there’s something like personality fit. So dominant people like to talk to others in a dominant way. And they also prefer the dominant chatbot. This is strange, but not surprising. So this is the same here. So that is what I would definitely recommend to you. I know it’s, also from a data security and privacy point of view, tricky to get some personal data, but at least something gender may matter, or age, or whatever information you get, may help you to target the communication better”. (Chatbot expert CE04)*

Rather than developing an adaptive dynamic chatbot with the aim of tailoring conversation style, it was recommended that different “versions” of the chatbot be developed which could then be deployed on the basis of initial user responses. Rather than this personalisation being an automatic process, one chatbot expert flagged this as potentially letting users have some control over the chatbot experience;

*“Beyond the content, you need to somehow think of how is this chatbot delivered to the user? And what is the ability of the user to somehow personalise? And I think that could make sense also to let people to configure a little bit at least the chatbot they prefer”. (Chatbot expert CE04)*

#### Positive & professional

In order to enhance engagement and support young adults to eat healthier, the preferred chatbot style was perceived by all stakeholder groups as professional, casual and friendly language, with minimal use of small-talk, and use of short sentences, with low language complexity:

*They’re expecting to talk to a professional. They’re not expecting to talk to someone that’s trying to talk like them… lay language, professional tone that’s friendly and positive.* (Dietary expert DE04)

*“.I would say obviously keep the sentences short and concise as possible. I think you showed at the very beginning of the interview, the two different types; one was more of a short form, one was a long form. I guess it depends on what information or content you’re communicating, but certainly I think with young adults attention can easily be lost. The sentence complexity should be one of the other top ones as well”. (Dietary expert DE03)*

*“…I would kind of limit the small talk and the jokes and things like that, because … if I’m there to look for information, I pretty much just want the answers straight away kind of thing…Just get the information across … succinctly and effectively” (Current website user U02)*.

Furthermore, many of the dietary experts and current website users voiced the importance of using a ‘positive tone’ and being constructive regarding nutrition and what people can or cannot eat. These discussions were closely related to content as well, in terms of messaging aligned with the short-term benefits of healthy eating (positive tone), as opposed to prevention of long-term health problems (negative tone). This was perceived as an important feature, as young people were reluctant and unlikely to engage with content using anything other than a positive tone;

*“I feel like constantly hearing the negative one mostly de-motivates… because it’s constantly… Like, you’re not… It feels like you’re not doing enough and the [positive] one feels more like you can improve even more. Which motivates me more to actually start eating healthier and actually put effort into it.” (Current website user U07)*.

*Really shifting the focus to a positive based message and a more immediate based outcome, like energy levels or mental wellbeing is a really positive way to sort of look at this population ‘cause chronic diseases for them well, they’re creeping up closer into middle adulthood, but they are still a fair way away. You want people to have a positive experience and not feel shamed or anything like that when they’re engaging with the health service. (Dietary expert DE04)*

Meanwhile, chatbot experts expressed a more fluid perspective on the importance of one ‘right’ tone and message. Instead, they emphasised embedding user assessments to determine ideal tone and messaging for the conversation.

### Chatbot messaging for dietary behaviour change

#### Education, training & enablement

Based on the examples provided to interviewees relating to BCW intervention functions, both dietary experts and users identified the key message types most important for dietary behaviour change were: education/informative (e.g., *did you know that bananas are high in potassium which can aid in reducing blood pressure?*), training (e.g., “*would you like to see the video to make this recipe?”*) and enablement (e.g., *“eating healthy doesn’t have to be expensive! We’ve created a meal plan with 7 breakfasts, 7 lunches and 7 dinners for less than $55. Want to see?“*). Specifically, these types of messages which have potential to show users how to build up their skills and knowledge (e.g., recipe videos, meal plans, tailored diet advice) were considered vital to help address barriers and support positive changes to eating habits:

*“I think then the training and enablement, I suppose it’s more like building up skills and showing them how you can build up their skills. I think that could be appealing for if they have identified that as a gap in their knowledge that they want to fill”. (Dietary expert DE05).*

*“I think the enablement one is optimistic. I think like my problem is that I have this idea that eating healthily causes quite a lot of… like it’s quite an… I’m trying to think of the word… bit of an effort because I don’t know the recipes and blah, blah, blah. This one, it gives me everything I need to hear; it gives you a meal plan so it can organize what you are going to eat and then it gives you how much it’s going to be. You can really justify it and makes me want to see more of it”. (Current website user U01)*

*“I really liked the training because sometimes when you read a recipe, it can be really daunting. Whereas I like to, I like visual aids to see how stuff are done”. (Current website user U03)*

*“I like the educational informative one. I think it’s good, kind of empowers you to understand why things are good for you. Obviously, you know bananas are healthy, but knowing that it’s high in potassium just gives you more information. And I guess makes you feel you’ve got more control of your diet and things like that”. (Current website user U04)*

Furthermore, while some chatbot experts generally felt that advice on messaging was outside their scope of practice, there was general consensus that healthy eating messages based on principles of training, education and enablement were also perceived as positive and empowering, as they were felt to foster more resilience and self-regulation.

## Discussion

The current study used a qualitative approach to explore potential utility and design of chatbots to increase young adults’ engagement with a purpose-built healthy eating website, ‘*No Money No Time’* [24, 25], and improve their diet quality over time. Interviews with 18 experts in dietary behaviour change in young adults, current website users, and experts in chatbot design yielded insightful information. This included details regarding the potential roles of a chatbot to support healthy eating, chatbot features and content, preferred style, language and messaging, as well as major factors influencing healthy eating in young adults. Findings can be used to guide the design of chatbots targeted and tailored to support healthy eating in young adults.

This study identified three potential roles of a chatbot to support healthy eating in young adults and eight suggested features/content to support each role. See Table 2 for potential use cases for each of the suggested features/content for a chatbot to support healthy eating. The proposed use cases provide examples based on capabilities of an integrated chatbot on a website, but features could be enhanced by use of a multi-channel approach (e.g., use of email automations to support a chatbot). It is important to note that the potential costs and reported time associated with development of the suggested features may not be feasible for researchers and developers. Therefore, an informed decision that weighs up advantages and disadvantages must be made with regards to timeline, budget and resources available.

**Table 2:** Potential use case examples based on suggested chatbot features to supporthealthy eating

Potential roles of chatbot	Suggested features/ content	Use cases	
		Context	Example interaction
R1. Improving healthy eating knowledge & facilitating discovery.	F1. Chatbot generated recommendations that are initiated by the chatbot to detail content which may be of interest to the user.	Based on recent browsing behaviour, chatbot will initiate conversations with recipes or articles the user is expected to like. For example:	Chatbot: *Hey there! Want to see some more microwave recipes?* *[yeah, sounds good] [nah]*
	F2. Triaging young people to information on the website (or externally) which would address current needs.	After completing an online Food Frequency Questionnaire on the site, the Chatbot can provide suggestions that promote healthy eating habits.	Chatbot: *Hi [name], would you like to see some recipes to help boost your healthy eating quiz score?* *[Take me there!] [I’m good thanks]*
R2. Reducing time as a barrier to engage in healthy eating.	F3. Nudge or behavioural prompts at critical moments	To initiate chatbot conversation around certain mealtimes.	Chatbot: *It’s almost dinner time! Tell me what food you have at home, and I’ll find some recipes for you.*
			User: *Chicken*
			Chatbot: *Here are 42 great chicken recipes. Include another ingredient to narrow it down*:
			*[insert recipe cards]*
	F4. Assist users to navigate the information on various pages within a healthy eating website.	Chatbot to respond to any related questions posed by the user.	User: *What are the best things to eat before exercise?* Chatbot: *Great question! Check out our blog article on this*:
			[Take me there]
R3. Providing support and Social engagement	F5. Enhanced interactivity	Chatbot to utilise different strategies to enhance interactivity (e.g., use of emojis)	Chatbot: *Send me an emoji of ingredients that you have at home and I will find some recipes for you?*
			User: *Tomato emoji sent*
			Chatbot: *Here are 52 great tomato recipes. Include another ingredient to narrow it down*:
			[insert recipe cards]
			User: *Chicken emoji sent* Chatbot: *Nice! 13 chicken and tomato recipes*
			[insert recipe cards]
	F6. Offer useful anonymous support to prevent any feelings of embarrassment and shame associated with topics and questions ‘discussed’.	Chatbot to respond to any sensitive questions from users in a supportive manner and direct to appropriate professional support when needed.	User: *What are the signs of an eating disorder?* Chatbot: *Different types of eating disorders have different symptoms, see our article here for more information.*
			[direct to article on website]
			Chatbot: *If you (or someone you know) would like professional support for eating or body image concerns. Please contact the following for free and confidential support*:
			[direct to appropriate professional support]
	F7. Facilitate user connection with content in a deeper more meaningful way because of the “social” conversational aspect of it.	Chabot to provide positive reinforcement and social comparison upon improvement to eating habits (determined by diet quality assessment tool embedded on site)	Chatbot: *Great job [John] you have improved your healthy eating score by XX points. Here’s a great big high five from the NMNT team!*
			[high five sticker]
			Chatbot: *Did you know that your healthy eating score is now in the top 20% of all males aged 18–24 years. Keep up the great work!*
	F8. Outreach adjunct to website - reaching users on the platforms that they are more likely to frequent (such as Facebook Messenger) or ‘bounce’ between email and website.	Chatbot to offer the option for users to opt in to email follow-ups and then users can be added to email automations.	Chatbot: *Would you like to receive personalised emails from us to help support your healthy eating journey?* *[Yes, sign me up!] [No thanks]*

Authenticity, personalisation, and effective and strategic development were identified as other important chatbot features in the current study. Suggestions included; ability of chatbots to mimic human conversation and recall past user information to achieve authenticity; initial screening and data collection by the chatbot to direct users to sections of the website to achieve personalisation; and a visually pleasing chatbot that picks up on keywords and option to minimise or close the chatbot, to ensure strategic development of the chatbot. These suggestions are consistent with the recommendations of Willmott et al. in their 2019 systematic review of weight management eHealth interventions in young adults [38]. This review noted that the use of persuasive technology elements and user interaction were significant predictors of intervention adherence, and subsequent intervention exposure and effect. Several of the recommended intervention strategies stemming from the review could be achieved using chatbot technology and are consistent with the themes identified in this study, including tailored or personalised feedback, contact with an interventionist, nudges, reminders and booster messages [38]. Notably, none of the eHealth interventions in the review used chatbots, instead using one or more of websites, text messaging, emails, wearable devices, apps, and social media. This highlights the novelty of using chatbots in the context of nutrition interventions, and of the formative work to inform this, such as the current study.

While there was some preference for an unconstrained chatbot (e.g., preference for human-to-human-like communication), findings suggest there was a greater preference towards a constrained (i.e., rule-based) chatbot:

*“I think what deters me is when it doesn’t give me the answer that I’m after and then it just keeps reverting back to the main menu instead of offering more assistance. But I think in, in this sort of situation, when there are sort of fixed options and sort of generalized knowledge.” (Current website user U02)*.

*“This is a very important factor that you do not open up … And sometimes you may allowfor open answers, but most of the successful chatbots predefine the structure of the dialogue to some degree. And that is what I definitely also would recommend to you to keep the dialogue simple and also [to have] partially predefined responses.” (Chatbot expert CE04)*.

Constrained chatbots are characterised by a well-structured design, simplicity in development, control, and implementation, which guarantees the quality and consistency of content delivery [4]. However, their inability to adapt to users’ inquiries and address unexpected questions renders them unsuitable for facilitating more authentic and intricate interactions with users [2]. On the contrary, unconstrained chatbots use Natural Language Processing (NLP) to simulate human-to-human communication, allowing for more natural dialogue and interaction which is perceived as more engaging [4, 39]. However, the cost and reported time associated with development of unconstrained chatbots may be a barrier to researchers and developers. In future research, the feasibility of utilising unconstrained chatbots may increase due to the growing accessibility of extensive healthcare datasets and advancements in technologies. For example, Large Language Models (LLMs) are a type of artificial intelligence designed to mimic human language processing abilities [40]. ChatGPT is a type of LLM model released by OpenAI (San Francisco, California) in 2018 which has already demonstrated huge potential in clinical and research healthcare scenarios [40]. Due to rapidly advancing nature of LLMs it would be useful to explore the potential feasibility of this technology within the context of dietary chatbots to support healthy eating for young adults.

In terms of chatbot style and language, the current study identified that tailoring, and positive and professional style and language are key. The suggestion of positive tone is consistent with qualitative findings from the Communicating Health Study, which aimed to understand how young adults use online media in relation to health [41]. The Communicating Health Study found that young adults themselves used positive, gain framed language in discussing ideas for future health campaigns. As noted by those interviewed in the current study, the use of negative tone (e.g., *did you know that eating too much red or processed meat can increase your risk of cancer?*) or message framing can be de-motivating and lead to inaction. The theme of tailoring in the current study referred to tailoring the communication style of the chatbot, for example by demographics such as age or gender, or by personality type such as a dominant communication style, as well as offering choice for young adults in terms of personalising the chatbot to enhance their user experience. The need to co-design with young adults and to tailor interventions to young adults is widely acknowledged in the literature [42–45]. The current study highlights that tailoring the intervention delivery and content is important, which is also a critical argument raised in the review by Willmott et al. [38]. Tailored communication styles can make conversations with chatbots more relatable as they resonate with user’s daily experiences in their daily social environment. Research has shown that chatbot designs with high perceived familiarity drive social presence (i.e., the feeling of warmth and human sociability perceived during human-chatbot interaction) and trust in the system, which are key drivers for interventions aiming to increase motivation and capability [46].

This study identified training (e.g., *would you like to see a video to make this recipe?*), enablement (e.g., *healthy eating doesn’t need to be expensive, we’ve created a budget meal plan, want to see?)* and education/informative (e.g., “*did you know bananas are high in potassium which can aid in reducing blood pressure?”*) as key messaging types that chatbots could use in the context of supporting healthy eating in young adults. This includes providing information, but also showing and telling how to use the information, the combination of which could support knowledge and skill building. Providing young adults with information without further instruction or advice on how to implement this to achieve healthy eating has been shown to be ineffective in previous nutrition interventions, regardless of delivery method [42]. Chatbots could be used to provide these types of messages in a stepped approach, guiding users to relevant sections of the website and content.

### Strengths and limitations

The methodological rigour was a key strength of the study and was achieved through (i) adoption of research and analytic methods appropriate to the aims; (ii) highly relevant background and qualifications of researchers with multi-disciplinary expertise and (iii) triangulation via use of different types of informants (exploring the topic from three stakeholder groups). In addition this study was strengthened by use of the Behaviour Change Wheel as a framework to guide the interview questions [23]. In terms of limitations, there was no opportunity for participants to comment and/or correct transcripts or provide feedback on findings. In addition, to help determine preferred chatbot message types, interviewees were provided with examples based on BCW intervention functions. This was done to ensure alignment with the BCW and to elicit more in-depth discussions, as interviewees may not be aware of the different message types. However, this limits openness in responses, as interviewees could not suggest their own message types. Recruitment of current website users from a single healthy eating website may have introduced selection bias and not be representative of the entire young adult population, as users may have more favourable eating habits and different responses/ideas compared to those who do not use the site. Despite this, previous research has shown a large proportion of young adult users of the No Money No Time site to have low diet quality [47]. Furthermore, the study sample was small and predominantly female, while current website users were mostly students, and the majority of current website users and dietary experts were from Australia, which may impact on the generalisability of findings. The proposed discussion for chatbot design and utility related to an integrated web-based chatbot. However, application of chatbots has evolved in recent years with greater capabilities and utilisation across different modalities (e.g., mobile applications). As such, none of the recent advancements are accounted for in these findings.

### Implications for research and practice

Chatbots could be an effective component within future web-based interventions aiming to support healthy eating in young adults. More broadly, the current findings could be applied to other *e*Health intervention modes including text messaging, and health behaviours such as physical activity, in terms of communication style, language and types of messaging. In this sense, the general set of requirements and features identified in the present study could provide researchers and practitioners with a shared frame of reference to assist in conceptualising how chatbots could add value to young adults in this context, and which features could be prioritised and introduced over time. The next step to enhance behaviour change support and engagement with the ‘No Money No Time’ website, will be to use this formative work to inform development and evaluation of a chatbot as part of a nutrition intervention strategy, and evaluate its’ acceptability and effectiveness. This addresses a gap in the literature and aligns with recent recommendations from a systematic review and meta-analysis of chatbots for lifestyle interventions [7] which highlighted the need evaluate different aspects of the chatbot user-experience (such as tone, personality, text-based vs. voice-based, and frequency of communication). As chatbot technology advances, and they are more widely used in health behaviour change initiatives, this will advance the field in terms of chatbot utility to support dietary behaviour change.

## Conclusion

To our knowledge, this is the first study to explore potential utility of a chatbot to support healthy eating in young adults. These findings may assist chatbot designers and dietary behaviour change researchers regarding potential chatbot roles, features, style and language and messaging in order to support healthy eating in young adults.

### Electronic supplementary material

Below is the link to the electronic supplementary material.

Supplementary Material 1: COREQ Checklist

Supplementary Material 2: Interview guide and supporting materials

Supplementary Material 3: Supplementary Tables

## Data Availability

The datasets used and/or analysed during the current study are available from the corresponding author on reasonable request.

## List of abbreviations

BCW: Behaviour Change Wheel
COREQ: Consolidated criteria for reporting qualitative research
