# Supplementary material for: Exploring the design and utility of an integrated web-based chatbot for young adults to support healthy eating: a qualitative study
Source: Int J Behav Nutr Phys Act. 2023 Oct 4;20:119. doi: 10.1186/s12966-023-01511-4 (PMC10548711; doi:10.1186/s12966-023-01511-4)
Supplement: Supplementary file 2 — Supplementary Material 2: Interview guide and supporting materials [file 12966_2023_1511_MOESM2_ESM.docx]

Additional File 2 – Interview guide and supporting materials

**Exploring the design and utility of an integrated web-based chatbot for young adults to support healthy eating: A qualitative study.**

Lee M Ashton, Marc TP Adam, Megan Whatnall, Megan E Rollo, Tracy L Burrows, Vibeke Hansen, Clare E Collins*.

***** Correspondence: clare.collins@newcastle.edu.au

**Contents**

| **Supporting information item** | **Page** |
| --- | --- |
| **Interview guide:** Interview questions asked by stakeholder groups | 2 |
| **Supporting Material 1:** Chatbot definition provide at commencement to each interview | 4 |
| **Supporting Material 2:** Message type and examples based on BCW intervention functions. | 5 |
| **Supporting Material 3:** Image to support question relating to content and style preferences for a chatbot. | 6 |

**Interview questions asked by stakeholder groups**

|  | **Interview questions** | | |
| --- | --- | --- | --- |
| **Behaviour Change Wheel (BCW) component** | **Experts in dietary behaviour change in young adults** | **Current website users** | **Experts in Chatbots design** |
| Intervention functions (red circle) | *1.How could a chatbot be used to address:*  *i) Capability barriers (barriers relating to knowledge/skills of healthy eating)*  *ii) Opportunity barriers (e.g., time, access, storage, peers)*  *iii) Motivation barriers (don’t want or need to eat healthy. Not developing a plan.)* | *1.Have you heard of (or seen) chat bots? and how would you feel about a chatbot guide you to support healthy eating?* | *1.How could a chatbot be used to address:*  *i) Capability barriers (barriers relating to knowledge/skills of healthy eating)*  *ii) Opportunity barriers (e.g., time, access, storage, peers)*  *iii) Motivation barriers (don’t want or need to eat healthy. Not developing a plan.)* |
|  | *2.What features of a chatbot are important for this group?* | *2.What features of a chatbot would make you interact with it?*  [If they say no I wouldn’t interact/ I don’t like them find out why] | *2.What features of a chatbot are important for young adults (18-35 years)? and what is possible in terms of conversation complexity (e.g. pre-defined answers, length of conversation).* |
|  | *3.What tone of messaging do you think would be more persuasive for young adults?*  Negative: *Did you know poor diet is linked to many chronic diseases*  *OR*  Positive: *Eating healthy can help to improve your energy levels and well-being* | *3.What tone of messaging would you prefer by a chatbot?*  Negative: *Did you know poor diet is linked to many chronic diseases*  *OR*  Positive: *Eating healthy can help to improve your energy levels and well-being* | *3.What tone of messaging would be more persuasive for young adults?*  Negative: *Did you know poor diet is linked to many chronic diseases*  *OR*  Positive: *Eating healthy can help to improve your energy levels and well-being* |
|  | **[Show BCW examples on PowerPoint screen and allow a few minutes to read – See supporting material 2 below]**  *4.What type of messaging would be more persuasive for young adults?* | **[Show BCW examples on PowerPoint screen and allow a few minutes to read - See supporting material 2 below]**  *4.What type of messaging would you prefer by chatbot?* | **[Show BCW examples on PowerPoint screen and allow a few minutes to read- See supporting material 2 below]**  *4.What type of messaging would be more persuasive for young adults?* |
|  | **[Refer to PowerPoint slide with Taxonomy of social cues – sub-section verbal- See supporting material 3 below]**  *5.What aspects of* ***content*** *and* ***style*** *are important for a chatbot to use when conversing with young adults about healthy eating?* | **[Refer to PowerPoint slide with Taxonomy of social cues – sub-section verbal - See supporting material 3 below]**  *5.What aspects of* ***content*** *and* ***style*** *are important for a chatbot to use when talking to you?* | **[Refer to PowerPoint slide with Taxonomy of social cues – sub-section verbal - See supporting material 2 below]**  *5.What aspects of* ***content*** *and* ***style*** *are important for a chatbot to use when conversing with young adults about healthy eating?* |
| Closing questions | *6.What else do you think may be important for chatbots to illicit positive dietary changes in young adults?* | *6.What else is important for you to improve diet habits* | *6.What else is important for chatbots to illicit positive dietary changes in young adults?* |
|  | *7.Now we have focussed on chatbots, what would you do differently if you were trying to (re-state the aims) by using automated email communication?* | *7.What makes interacting with a chatbot different from receiving an automated email? Does it matter? Why? Would you prefer interacting with a chatbot rather than email? Why?* | *7.What makes designing a chatbot different from creating automated emails? What would you do differently for a chatbot rather than designing automated emails?* |

**Supporting Material 1: Chatbot definition provide at commencement to each interview**

1. Image provided on PowerPoint


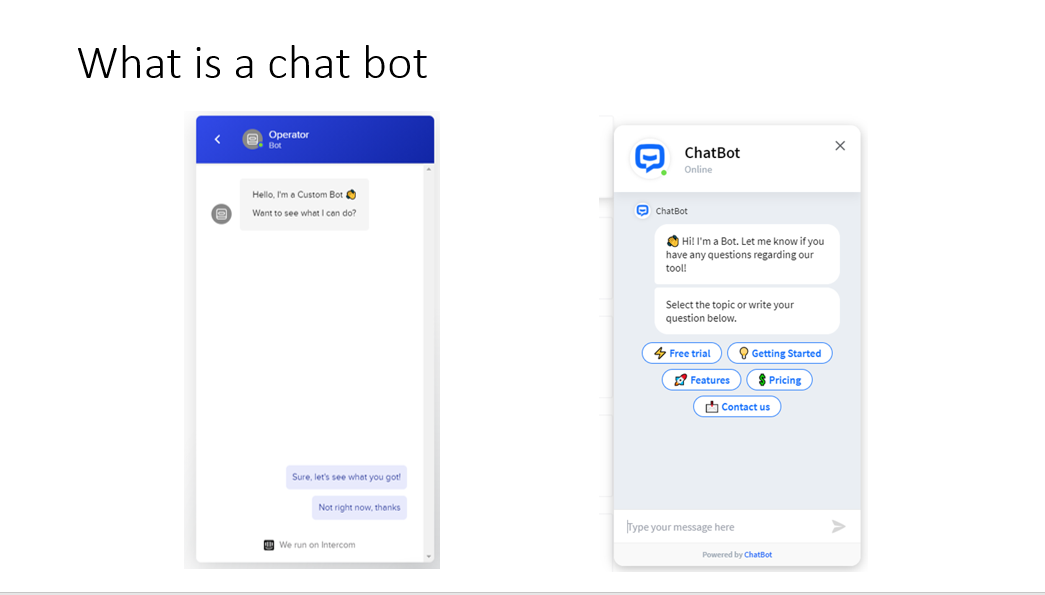


2. Accompanying script: *Chatbot is simply a computer system intended to converse with a human. It has the ability to engage in “small talk” and casual conversation BUT usually just* ***1 sentence, 1 response, its simplistic conversations.*** *You may have come across a chatbot on a website before, in many cases you enter a website and chat bot pops up to help assist you on the site. The example on the left is a Closed conversation – can only select pre -determined responses then the conversation will filter down until you arrive at the answer The example on the right is a bit more advanced and features Open and closed Qs – uses natural language processing to help choose appropriate course of action*

**Supporting Material 2:** Message type and examples based on BCW intervention functions.

**Context:** provided to interviewees via PowerPoint to support question: “*What type of messaging would be more persuasive for young adults?”*


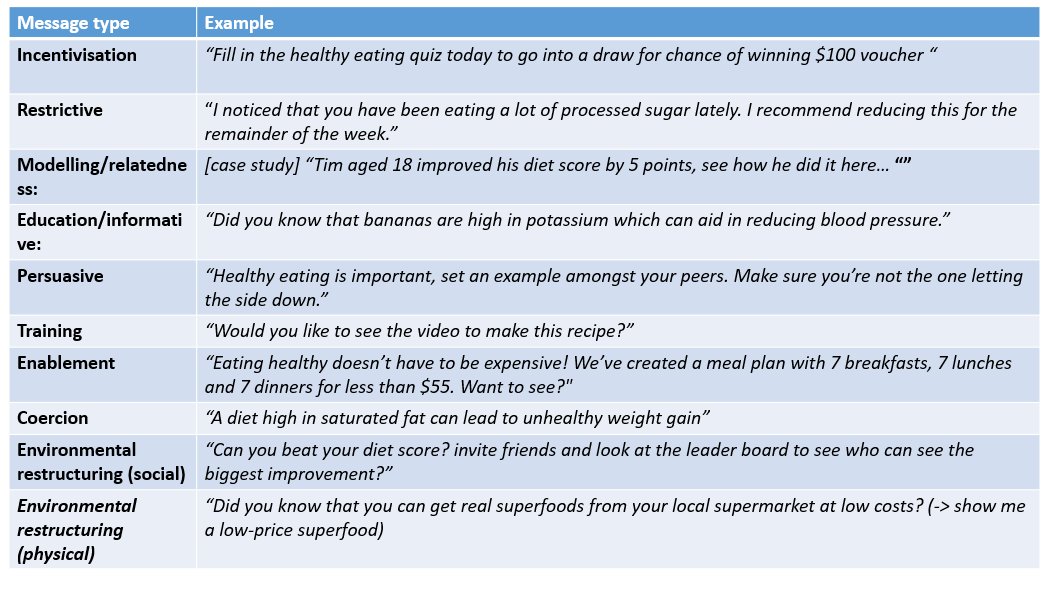


**Supporting Material 3:** Image to support question relating to content and style preferences for a chatbot.

**Context:** provided to interviewees via PowerPoint to support question: “*What aspects of* ***content*** *and* ***style*** *are important for a chatbot to use when conversing with young adults about healthy eating?*


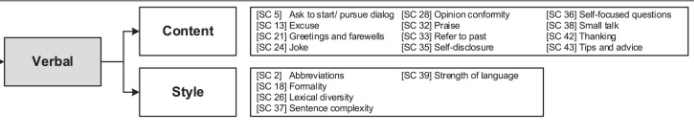


Image reference: taxonomy of social cues for conversational agents. Image obtained from: Feine J, Gnewuch U, Morana S, Maedche A. A taxonomy of social cues for conversational agents. International Journal of Human-Computer Studies. 2019;132:138-61.(1)

REFERENCES

1. Feine J, Gnewuch U, Morana S, Maedche A. A taxonomy of social cues for conversational agents. International Journal of Human-Computer Studies. 2019;132:138-61.
