# Supplementary material for: Exploring the design and utility of an integrated web-based chatbot for young adults to support healthy eating: a qualitative study
Source: Int J Behav Nutr Phys Act. 2023 Oct 4;20:119. doi: 10.1186/s12966-023-01511-4 (PMC10548711; doi:10.1186/s12966-023-01511-4)
Supplement: Supplementary file 3 — Supplementary Material 3: Supplementary Tables [file 12966_2023_1511_MOESM3_ESM.docx]

Additional File 3 – Supplementary Tables

**Exploring the design and utility of an integrated web-based chatbot for young adults to support healthy eating: A qualitative study**

Lee M Ashton, Marc TP Adam, Megan Whatnall , Megan E Rollo, Tracy L Burrows, Vibeke Hansen, Clare E Collins*.

***** Correspondence: clare.collins@newcastle.edu.au

**Contents**

| **Supporting information item** | **Page** |
| --- | --- |
| **Supplementary Table 1.** Background of interview participants. | 2 |
| **Supplementary Table 2.** Potential roles of a chatbot to support healthy eating in young adults | 4 |
| **Supplementary Table 3**. Other general Chatbot features to support healthy eating. | 7 |
| **Supplementary Table 4:** Suggested Chatbot style and language to support healthy eating in young adults | 10 |
| **Supplementary Table 5:** Suggested Chatbot messaging to support healthy eating in young adults | 12 |

**Supplementary Table 1:** Background of interview participants.

| **Stakeholder** | **ID** | **Background & Experience** | **Age Cat** | **Country of residence** | **Sex** |
| --- | --- | --- | --- | --- | --- |
| Experts in dietary behaviour change in young adults. | DE01 | Academic. PhD, BNutrDiet. Accredited Practising Dietitian. 40+ publications. Research focus on behaviour change with young adults. | 25-29 years | Australia | Female |
|  | DE02 | Academic. PhD, BNutrDiet. Accredited Practising Dietitian. 110+ publications. Research focus on digital health and behaviour change with young adults. | 35-39 years | Australia | Female |
|  | DE03 | Academic. PhD. B.Bus. 10+ publications. Research focus of weight management in young adults | 25-29 years | Australia | Female |
|  | DE04 | Academic. PhD, BNutrDiet. Accredited Practising Dietitian. 55+ publications. Research focus on digital health and behaviour change with adolescents and young adults. | 30-34 years | Australia | Female |
|  | DE05 | Accredited Practising Dietitian. BNutrDiet. Business owner. | 20-24 years | Australia | Female |
|  | DE06 | Academic. PhD, BNutrDiet. Accredited Practising Dietitian. 9 publications. | 25-29 years | Australia | Female |
| Current website users | U01 | Employment status: Student. Main occupation field: Health and Medical. | 20-24 years | Australia | Female |
|  | U02 | Employment status: Working full-time paid employment. Main occupation field: Engineering. | 25-29 years | Australia | Female |
|  | U03 | Employment status: Student. Main occupation field: Education. | 20-24 years | Australia | Female |
|  | U04 | Employment status: Student. Main occupation field: Hospitality, travel and tourism. | 20-24 years | Australia | Female |
|  | U05 | Employment status: Working full-time paid employment. Main occupation field: Management. | 20-24 years | Australia | Female |
|  | U06 | Employment status: Student. Main occupation field: Engineering. | 30-34 years | Poland | Female |
|  | U07 | Employment status: Student. Main occupation field: N/A | 20-24 years | Netherlands | Male |
| Experts in chatbot design | CE01 | Academic. PhD. MSc. BSc. 6 publications. Research focus on AI-based process automation and augmentation (e.g., chatbots) | 30-34 years | Germany | Male |
|  | CE02 | Academic. PhD. 5+ publications. Research focus on Human Computer Interaction, Interaction Design and User Experience (UX). | 35-39 years | New Zealand | Female |
|  | CE03 | Academic. PhD. 15+ publications. Research focus on machine learning for health applications. | 35-39 years | Australia | Male |
|  | CE04 | Academic. Professor. 430+ publications. Research focus on: Information Systems and Human-Computer Interaction | 45-49 years | Germany | Male |
|  | CE05 | Academic. PhD. 35+ publications. Research focus on Human Computer Interaction and conversational agents. | 40-44 years | USA | Male |

BNutrDiet = Bachelor of Nutrition and Dietetics. B.Bus.= Bachelor of Business. BSc = Bachelor of Science. MSc = Master of Science.

| **Supplementary Table 2:** Potential roles of a chatbot to support healthy eating in young adults | | |
| --- | --- | --- |
| **Role** | **Suggested chatbot features/content relating to role** | **Supporting quotes (stakeholder type, ID)** |
| R1: Improving healthy eating knowledge & facilitating discovery | - F1. Chatbot generated recommendations that are initiated by the chatbot to detail content which may be of interest to the user. | *“…maybe you have recipes there and people visit that section of the website and you can open the chat bot and say, “Hey I see you are looking for recipes with... Here's some of my favorite suggestions. If you want to learn more click here...”(Chatbot expert CE01)*  *…you could have the chatbots saying like, let's say again, you're looking at like, healthy, vegetarian recipes or something like on the chatbot and it comes up with a recipe. But then it might say to you, “oh, now that you looked at, would you like to see also blah” ... It could kind of sneak in, introduce some other good topics that you think might be of interest or that you might want to just promote. (Chatbot expert CE02).* |
|  | - F2. Triaging young people to information on the website (or externally) which would address current needs. | *Like they could pinpoint someone's lack of knowledge or lack of skills and then maybe direct towards that resource that might help them fill that gap. (Dietary expert DE05)* |
| R2. Reducing time as a barrier to engage in healthy eating | - F3. Nudge or behavioural prompts at critical moments | *I think if the chat bots there to maybe give some nudges or behavioural prompts or provide some recipes in real time to be a real advantage for young adults that are busy or think that they don't have time to look at recipes or potentially get home and they're too busy and they forget and then end up ordering out or getting something delivered. (Dietary expert DE03)* |
|  | - F4. Assist users to navigate the information on various pages within a healthy eating website. | *I think it would be really good if, if I asked a question and it took me exactly to that spot for my answer. (Current website user U01)* |
| R3. Support and social engagement | - F5. Enhanced interactivity | *The ones that are available from government or public health organizations are just pretty static and like text-based boring...they're all written at a university level. Just having that extra elements on the pages and making them more interactive I think is a really positive step. (Dietary expert DE04)* |
|  | - F6. Offer useful anonymous support to prevent any feelings of embarrassment and shame associated with topics and questions 'discussed'. | *I guess the first thing that came to mind was that I think both chatbots and conversational agents offer a viewer of secrecy, the same type of viewer secrecy that we have when we search for random things on Google. I think there is quite a bit of stigma when it comes to certain, for example, types of eating disorders. The first thing that came to mind is that something like this would perhaps make it safer for some young adults to get information, get feedback with regards to some sensitive topics related to healthy and unhealthy eating. (Chatbot expert CE03)* |
|  | - F7. Facilitate user connection with content in a deeper more meaningful way because of the "social" conversational aspect of it. | *I really do think that to trigger behavior change, you need support, you need a network, and you need a good sense of community. I think technology can definitely provide those as well. And so rather than having a standalone chatbot that a person interacts with, I think that in order to trigger behavior change, you would probably want the chatbot with some social aspect, whether it's comparison, whether is getting encouragement and support from your social network, and that's connected in some way to the chatbot. (Chatbot expert CE03)* |
|  | - F8. Outreach adjunct to website - reaching users on the platforms that they are more likely to frequent (such as Facebook Messenger) or ‘bounce’ between email and website. | *They don't need to go on a website or look for some information. The information comes to them. (Chatbot expert CE01)*  *If you aim for engagement, then you need to use one of the channels that people use anyways. (Chatbot expert CE01)*  *“You have maybe somehow our connection to this machine because you're having a chat. And when the bot at the end of your chat asks you, “Hey, that was nice, I would like to stay in touch with you. Could you please … if you want to give me your email and I will send you interesting content”. And this is one way that companies use it to, to get email addresses. And maybe this could also be interesting that the bot is not just one way to increase engagement within the bot, but also to recruit people who are interested for, for these automated emails. And then in the emails you can say, “Hey, we've recently updated our chat bot. It now has interesting recipes, come here visited again so you can try to...”. (Chatbot expert CE01)* |

**Supplementary Table 3:** Other general Chatbot features to support healthy eating.

| **Chatbot features** | **Detail** | **Supporting quotes (stakeholder type, ID)** |
| --- | --- | --- |
| Authenticity | - Mimic human conversation allowing for open conversations. | *At the motivational level...it depends really on I guess the individual, as to whether a chatbot can increase motivation and whether it comes through is genuine and authentic, cause you're not really talking to a real person. Again, [it depends on] the sophistications in technology. (Dietary expert DE03)* |
|  | - Hybrid chatbot – capable of simple responses but referred to human contact where possible. | *… option to ask a question to a human if it doesn't understand your question, that's usually something I like in a chatbot (Current website user U07)*  *This is where you have a chatbot as a single contact. And if the chatbot doesn't know how to move on, it hands over to a human. But this is also something which I could imagine in this foods context where you have some person that really gives you personal advice in case you don't trust the chatbot, or you want to talk to a human. So that could be a feature where you just have a phone call with someone or you hand over to an instant message chat with real human. (Chatbot expert CE04)* |
|  | - Ability to remember past user responses. | *For example, if the chatbot asks Jimmy to enter how many calories they ate, and Jimmy enters, "I ate 1000 calories," then certain bits of information might be easy to capture, because you would be looking for maybe, let's say ... In terms of calories, you would be looking for a number and the word 'calorie' nearby. And then you could just save that in a database, and then the chatbot, the next session the chatbot might say, "Oh, yesterday you ate ..." fill in the blank, pull the value from the database. (Chatbot expert CE03)* |
| Personalisation | - Use chatbot as data collection tool to target needs and characteristics of users and tailor responses. | *If there's some way of personalizing it a bit to the individual by perhaps asking them a few of those more closed ones on the left-hand side, to get some information about them to then be able to answer the questions in a more personalized way. I think that would make it more engaging and useful for young adults as well. (Dietary expert DE02)*  *So maybe if there was certain foods that you liked or didn't like, or if you're vegetarian, or an amount of time that you wanted to spend cooking. The amount of money you wanted to spend on cooking for the day. Maybe how many meals you cook a day or a week or whatever. (Current website user U04)*  *The more you know about the user, the better you can trigger behavior change. And that is why this data collection is still super important. (Chatbot expert CE04)* |
|  | - Initial ‘screening’ to establish needs | *The screening could be something really simple as first as like “what are you here for today?” kind of thing and then it has like a few different options… so as sort of a screening system that I guess identifies that person's maybe stage of change or where they're at, and then that would then influence whether the bot has like a negative or a positive tone or a mixture of both. (Chatbot expert CE02)* |
|  | - Link with website data | *If you see that the person is on the website in the section where you talk about sugar, and then there's the chat bot pops up and tells you, “Hey here's some cool information, you didn't know about sugar”. And I think this is also personalization based on what the user did, but you don't have to know his or her habits. (Chatbot expert CE01)* |
| Effective & strategic development. | - Visually pleasing and eye-catching interface (use of avatar, animations, icons etc) | *I know sometimes on websites they have like a little human, like a graphical person that pops up, like a little animation and it makes it a little bit more personable. Yeah, that's kind of handy I guess. Yeah. (Current website user U02)* |
|  | - Picks up keywords and sends them to the right information. A simple ‘closed’ keyword functionality was seen as good starting point. | *I think what deters me is when it doesn't give me the answer that I'm after and then it just keeps reverting back to the main menu instead of offering more assistance. But I think in, in this sort of situation, when there are sort of fixed options and sort of generalized knowledge.. (Current website user U02)*  *This is a very important factor that you do not open up … And sometimes you may allow for open answers, but most of the successful chatbots predefine the structure of the dialogue to some degree. And that is what I definitely also would recommend to you to keep the dialogue simple and also [to have] partially predefined responses. (Chatbot expert CE04)* |
|  | - Option to minimise/ close chatbot | *So if it's like show up only once and then you can like minimise that or open it again, or, you know, there is an option to open it or close, but it's not like it's going to be showing up every five minutes or something. (Current website user U06)* |

**Supplementary Table 4:** Suggested Chatbot style and language to support healthy eating in young adults

| **Style and Language** | **Detail** | **Supporting quotes (stakeholder type, ID)** |
| --- | --- | --- |
| Tailoring | - Tailor communication style based on basic demographics (e.g., age and gender) | *We did some research on personality and chatbots. And we looked at how different personalities of users may be influenced if the chatbot is talking more dominant or less dominant with the user. And there's something like personality fit. So dominant people like to talk to others in a dominant way. And they also prefer the dominant chatbot. This is strange, but not surprising. So this is the same here. So that is what I would definitely recommend to you. I know it's, also from a data security and privacy point of view, tricky to get some personal data, but at least something gender may matter, or age, or whatever information you get, may help you to target the communication better. (Chatbot expert CE04)* |
|  | - Enable users to personalise chatbot experience | *Beyond the content, you need to somehow think of how is this chatbot delivered to the user? And what is the ability of the user to somehow personalize? And I think*  *that could make sense also to let people to configure a little bit at least the chatbot they prefer. (Chatbot export CE04)* |
| Positive & professional | - Use professional, casual and friendly language | *They're expecting to talk to a professional. They're not expecting to talk to someone that's trying to talk like them... lay language, professional tone that's friendly and positive. (Dietary expert DE04)* |
|  | - Use of short sentences with low language complexity | *.I would say obviously keep the sentences short and concise as possible. I think you showed at the very beginning of the interview, the two different types; one was more of a short form, one was a long form. I guess it depends on what information or content you're communicating, but certainly I think with young adults attention can easily be lost. The sentence complexity should be one of the other top ones as well. (Dietary expert DE03)*  *…I would kind of limit the small talk and the jokes and things like that, because … if I'm there to look for information, I pretty much just want the answers straight away kind of thing…Just get the information across … succinctly and effectively (Current website user U02)* |
|  | - Positive tone | *I feel like constantly hearing the negative one mostly de-motivates... because it's constantly... Like, you're not... It feels like you're not doing enough and the [positive] one feels more like you can improve even more. Which motivates me more to actually start eating healthier and actually put effort into it. (Current website user U07)*  *Really shifting the focus to a positive based message and a more immediate based outcome, like energy levels or mental wellbeing is a really positive way to sort of look at this population ‘cause chronic diseases for them well, they're creeping up closer into middle adulthood, but they are still a fair way away. You want people to have a positive experience and not feel shamed or anything like that when they're engaging with the health service. (Dietary expert DE04)*  *I think for me the positive tone would be more persuasive for young people. I think they're like linking poor diet with chronic disease; doesn't work that well for young people because they feel a little bit invincible. It's not happening to them right now. I think if you're trying to find motivation, like you need to be looking at changes that will then, they will see immediately. The positive tone of like increasing your energy levels and wellbeing, you can see that immediately if you make those changes versus stopping chronic disease 40 years down the track, that's not that motivating for a young person. (Dietary expert DE05)* |

**Supplementary Table 5:** Suggested Chatbot messaging to support healthy eating in young adults

| **Messaging** | **Detail** | **Supporting quotes (stakeholder type, ID)** |
| --- | --- | --- |
| Education, training & enablement  **Training:** *“Would you like to see the video to make this recipe?”*  **Enablement:** *"Eating healthy doesn’t have to be expensive! We’ve created a meal plan with 7 breakfasts, 7 lunches and 7 dinners for less than $55. Want to see?"*  **Education/informative:** *Did you know that bananas are high in potassium which can aid in reducing blood pressure.* | - Show users how to build up skills & knowledge (e.g., recipe videos, meal plans, tailored diet advice etc) | *I think then the training and enablement, I suppose it's more like building up skills and showing them how you can build up their skills. I think that could be appealing for if they have identified that as a gap in their knowledge that they want to fill. (Dietary expert DE05)*  *I think the enablement one is optimistic. I think like my problem is that I have this idea that eating healthily causes quite a lot of… like it's quite an… I'm trying to think of the word… bit of an effort because I don't know the recipes and blah, blah, blah. This one, it gives me everything I need to hear; it gives you a meal plan so it can organize what you are going to eat and then it gives you how much it's going to be. You can really justify it and makes me want to see more of it. (Current website user U01)*  *I really liked the training because sometimes when you read a recipe, it can be really daunting. Whereas I like to, I like visual aids to see how stuff are done. (Current website user U03)*  *I like the educational informative one. I think it's good, kind of empowers you to understand why things are good for you. Obviously, you know bananas are healthy, but knowing that it's high in potassium just gives you more information. And I guess makes you feel you've got more control of your diet and things like that. (Current website user U04)* |
